# Objective monitoring of postpartum uterine activity: a systematic scoping review

**DOI:** 10.3389/fmed.2026.1703494

**Published:** 2026-03-05

**Authors:** Phebe B. Q. Berben, Marion W. E. Frenken, Annemarie F. Fransen, Eugenie J. L. G. Delvaux, Myrthe van der Ven, M. Beatrijs van der Hout-van der Jagt, S. Guid Oei, Judith O. E. H. van Laar

**Affiliations:** 1 Department of Obstetrics and Gynecology, Máxima Medical Center, Veldhoven, Netherlands; 2 Department of Electrical Engineering, Eindhoven University of Technology, Eindhoven, Netherlands; 3 Eindhoven MedTech Innovation Center (e/MTIC), Eindhoven, Netherlands; 4 Department of Obstetrics and Gynecology, Zuyderland Medical Center, Sittard-Geleen, Netherlands; 5 Department of Science and Medical Innovation, Máxima Medical Center, Veldhoven, Netherlands; 6 Department of Biomedical Engineering, Eindhoven University of Technology, Eindhoven, Netherlands

**Keywords:** myometrial activity, postpartum, third stage of labor, uterine contraction (MeSH), uterine monitoring (MeSH)

## Abstract

**Objective:**

To minimize risks of postpartum hemorrhage, understanding normal postpartum uterine activity is essential. This scoping review summarizes literature on postpartum uterine activity to provide insight into uterine activity (patho)physiology, characteristics of objective postpartum uterine monitoring methods and the effect of uterotonics on postpartum uterine activity.

**Data sources:**

A systematic search was conducted in PubMed, Embase and Cochrane in August 2024 and repeated in January 2025. No filter restrictions were applied. Systematic article selection was performed by two independent reviewers.

**Eligibility criteria:**

Articles were included if study participants were ≥ 18 years old and had external tocodynamometry (TOCO), intrauterine pressure catheter (IUPC) and/or electrohysterography (EHG) monitoring postpartum. Reviews, case reports, conference papers, technical modeling methods, guidelines, grey literature and duplicates were excluded, as were articles describing non-pregnant, non-human, non-labor studies and intrapartum studies.

**Study appraisal and synthesis methods:**

The Newcastle-Ottawa Quality Assessment Scale and the revised Cochrane risk-of-bias tool were conducted to assess study quality. Data was collected and systematically organized by two independent reviewers.

**Results:**

Twenty-nine articles were included after evaluation of 5,826 articles. Data analysis included 23 articles (IUPC n = 16, EHG n = 6 and TOCO n = 1) after risk of bias selection. Uterine contraction frequency without uterotonics ranges between 2.4 and 2.8 contractions per 10 min and between 3.7 and 4.6 contractions per 10 min with oxytocin, both decreasing over time. Normal baseline activity after childbirth is ≤ 15 mmHg and normal uterine intensity varies between 51–58 mmHg and 336–396 Montevideo Units. Studies conducted prior to 2020, measuring uterine activity with IUPC or TOCO, report no significant correlation between uterine activity and total blood loss. However, a small study using EHG conducted in 2024, cautiously suggests a positive relationship. This review highlights the need for a uniform and objective method to monitor postpartum uterine activity to adequately investigate the impact of different uterotonics on uterine activity.

**Conclusion:**

The (patho)physiology of postpartum uterine activity remains largely underexplored. EHG shows potential in enhancing our understanding of normal postpartum uterine activity as well as in postpartum hemorrhage recognition and prediction.

## Introduction

Uterine contractions during the postpartum period are crucial for placental expulsion and prevention of excessive blood loss by compressing spiral arteries (1). Postpartum hemorrhage (PPH) is traditionally defined as blood loss ≥ 500 mL within 24 h after vaginal delivery (VD) or blood loss > 1,000 mL after cesarean delivery (CD) (2). The reported rates of PPH after VD were 17% for total blood loss (TBL) ≥ 500 mL and 6% for TBL ≥ 1,000 mL, globally (3, 4). PPH remains the leading cause of maternal mortality and morbidity worldwide (5, 6).

The primary causes of PPH are categorized into tone (uterine atony), trauma (genital tract or surgical injury), tissue (retained placental tissue) and thrombin (coagulopathies) (7). Uterine atony is responsible for up to 80% of PPH cases, whereas retained placental tissue is the main cause of severe PPH (TBL ≥ 1,000 mL) (8, 9). Induced labor and prior severe PPH are known risk factors for PPH (10). Furthermore, uterine atony is more likely to occur in case of multiple gestations and fetal macrosomia, whereas retained placental tissue is often associated with previous uterine surgery or placental abnormalities (10–12). However, PPH may occur even without risk factors, making it an important issue for all laboring women (13). Therefore, prophylactic uterotonics after childbirth are recommended for all women since the year 2000. This is in accordance with the guidelines of the World Health Organization (14). Despite prophylactic treatment, PPH rates due to uterine atony are still rising (7).

Research on PPH has primarily focused on identifying risk factors for uterine atony and retained placental tissue, as well as on determining the optimal treatment methods (15). The underlying mechanism of both uterine atony and retained placental tissue may be attributed to inadequate contractile function of the myometrium (1, 16). Understanding the physiology of postpartum uterine activity (UA) (e.g., frequency, duration and intensity of contractions) is crucial for comprehending the pathophysiology of various etiologies of PPH. This insight can contribute to an improved and expedited recognition of an impeding PPH and it may improve prevention and successful management of PPH.

However, there is still limited research available regarding the physiology and pathophysiology of postpartum UA. The lack of postpartum UA data is largely due to technical limitations in objective monitoring methods suitable for use in clinical practice. Current techniques for continuous monitoring of UA during labor include external tocodynamometry (TOCO), intrauterine pressure catheter (IUPC) and electrohysterography (EHG). TOCO is the most widely utilized uterine monitoring method, although it is prone to inaccuracy and its performance is negatively influenced by maternal movements and obesity (17, 18). IUPC is considered the gold standard for intrapartum uterine monitoring. While IUPC can improve signal quality as compared to TOCO, it is invasive and associated with rare but serious risks such as uterine perforation (19, 20). EHG is a promising non-invasive technique that is more accurate and reliable in detecting contractions compared to TOCO during labor (21, 22). Furthermore, the electrophysiological signals are independent of body mass index (BMI) and maternal movements (22, 23). While the abovementioned monitoring methods have extensively been studied for intrapartum use (21, 24–27), their added value for postpartum use remains largely unknown.

This scoping review aims to provide an overview of the existing literature on postpartum UA monitoring to increase our understanding about the (patho)physiology of postpartum UA, and to highlight key themes and knowledge gaps in this field. It seeks to answer the main research question: what is known about postpartum UA? This main question is supported by sub questions:

1. What is known about the physiology of postpartum UA?
2. What is known about the pathophysiology of postpartum UA?
3. Which methods can be used to monitor postpartum UA?
4. What is the effect of uterotonic medication on postpartum UA?

## Methods

### Study design

The approach to guide this scoping review followed the six-step methodological framework outlined by Arksey and O’Malley. Extensions by Levac et al., and Peters et al. were incorporated (28–30). In addition, the PRISMA-ScR checklist was followed in designing and conducting of this scoping review (31). The protocol was preregistered on Open Science Framework on 16-08-2024, before literature search and data extraction was performed (32). The protocol was modified with the addition of the risk of bias assessment on 21-02-2025. Although a risk of bias assessment was not required in scoping reviews, including this assessment enhanced the transparency of the results while ensuring that no interpretative conclusions were drawn from low-quality literature.

### In- and exclusion criteria

Articles were selected when meeting the following inclusion criteria: describing studies including women ≥ 18 years old and using the following uterine monitoring methods: TOCO, IUPC and/or EHG. Outdated monitoring methods (e.g., Lorand tocograph), which preceded the currently used techniques, were not included. Exclusion criteria were: literature review, case reports, conference papers, technical modeling methods, guidelines, grey literature, duplicates, non-pregnant, non-human, non-labor and intrapartum monitoring. As the available literature was expected to be limited, no filter restrictions were applied.

### Information sources, search strategy and study selection

Systematic searches were performed by a librarian in the following databases: PubMed, Embase and Cochrane Library on 13-08-2024. The following terms were used (including synonyms and closely related words) as index terms or free-text words: “uterine monitoring” (e.g., uterine contraction, uterine inertia, electromyography) and “postpartum period” (e.g., labor stage, third). The full search strategy for all databases is available in Supplementary File 1. Duplicated articles were excluded. Identified articles were imported to Rayyan QCRI, a systematic literature review management web-based program (33).

Systematic article selection was independently completed in three stages by two reviewers (PB and MF). After each screening stage, disagreements were discussed until consensus was reached. In the first stage, article selection was performed by reading title and abstract. In the second stage, keywords were identified, and in case of doubt, full-text was read. Articles not in English or Dutch were translated. Finally, reference lists of selected articles were checked for possible additional articles. After completion of the full-text screening, the search was repeated on 21-01-2025 to capture any newly published articles and avoid missing relevant articles.

### Data extraction and synthesis

Data was collected and systematically organized by two independent reviewers (PB and MF) using a standardized table. Discrepancies between reviewers were resolved through discussion. In addition, uterine contraction frequencies (UCF) which were reported per hour or per 15 minutes (min), were converted to UCF per 10 min to facilitate comparison of results. In addition, values for UA intensity reported in Torr were converted to mmHg.

### Assessment of risk of bias

The Newcastle-Ottawa Quality Assessment Scale (NOS) for cohort studies and the revised Cochrane risk-of-bias tool (RoB 2) for randomized controlled trials (RCTs) were applied (34, 35). Quality assessments were independently assessed by two reviewers (PB and MF). Disagreements were resolved through discussion. Cohort studies were qualified as low, moderate or high quality (i.e., high, moderate and low risk of bias) with the following overall points: <4, 5–6 and 7–9, respectively. The NOS and RoB 2 tools are presented in Supplementary File 2.

## Results

### Article selection

**Figure 1:**
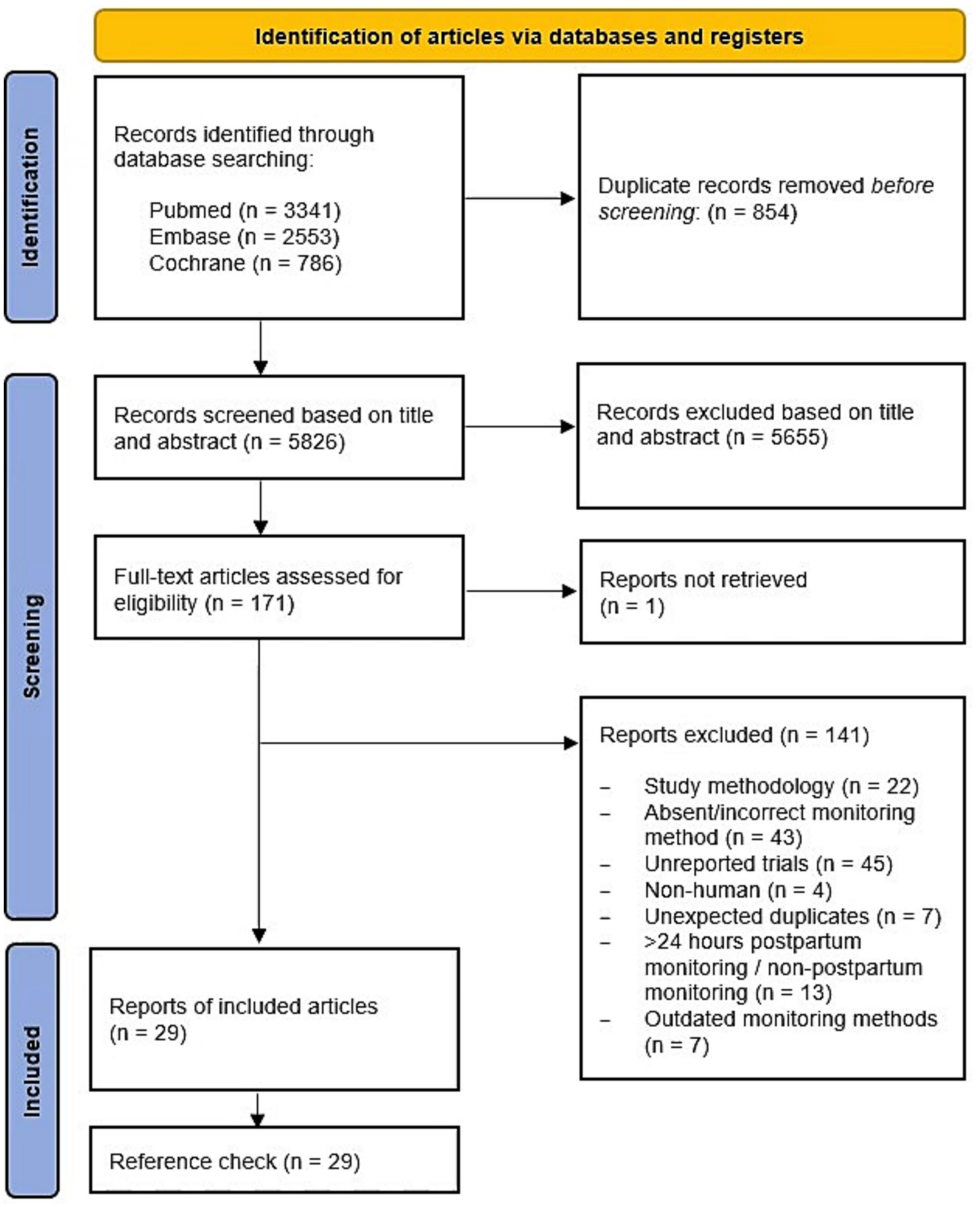
The PRISMA-ScR flow diagram of the article selection [following the template of Tricco et al. (31)].

A total of 5,826 articles were screened after removing 854 duplicates. Due to lack of specific titles and the frequent absence of abstracts, 171 articles were included for full-text reading. Finally, 29 articles were eligible for inclusion in this study. Figure 1 shows the PRISMA 2020 flow diagram of the article selection.

### Study characteristics

The study characteristics of all included articles are presented in Table 1. The 29 articles describe 28 studies, conducted in three different continents and eleven different countries. Two articles of Egarter et al. described the same underlying study. The majority of studies were conducted in Europe (n = 16), followed by Asia (n = 6) and North-America (n = 7). Furthermore, 15 of 29 articles were published before the year 2000. Regarding the study design, 83% were cohort studies, whereas 17% were RCTs. The sample sizes varied between 5 and 91 participants.

**Table 1:** Study characteristics of all included articles.

First author, year, country	Study design	Primary study objectives	*N*	Population description	Monitoring description	Uterine activity assessment	Results
IUPC							
Marx, 1978, USA (63)	Prospective cohort study	Effect of different anesthesia on postpartum UA	20	*Inclusion:* Healthy pregnant women Normal spontaneous delivery under nitrous oxide and oxygen anesthesia *Exclusion:* not described *Postpartum medication:* 10 mU oxytocin (Pitocin) iv was given at various depths of anesthesia *Procedure:* After placental expulsion anesthesia was induced with thiopental and maintained with nitrous oxide and oxygen (4:6) an 0.1% succinylcholine infusion Halothane anesthesia or enflurane anesthesia with 6 L oxygen in a non-rebreathing system, given 2 contractions after IUPC placement Continued anesthesia with nitrous oxide and oxygen mixture % PPH not described	Csapo microballoon with Statham P-1000A pressure transducer During ± 45 min after placental expulsion: (2 contractions before anesthesia vs. the remained time with effect of anesthesia) Timing with regard to placental expulsion: not described	Frequency, intensity and duration of UC. UA in MVU. UA analysis method: not described	Low concentrations of halothane (0.5 vol%) and enflurane (1.0 vol%) do not affect postpartum uterine activity. Higher concentrations (0.75 vol% halothane or 1.5 vol% enflurance) decreased uterine frequency and intensity
Marx, 1979, USA (62)	Prospective cohort study	Effect of four doses of ketamine on postpartum UA	22	*Inclusion:* Labor started after midnight, at least 5 h after the last food or fluid ingestion Received oral antacid every 3 h Spontaneous VD with a pudendal block or nitrous oxide-oxygen analgesia *Exclusion:* not described *Postpartum medication:* 10 mIU oxytocin (Pitocin) iv was administrated when the effect of ketamine on UA was disappeared, followed by a continuous infusion of 1.75 mL/min (*n* = 9) *Procedure:* Ketamine 25 mg iv or ketamine 50 mg iv or ketamine 75 mg iv or ketamine 100 mg, 10 min after IUPC placement with continuous oxygen administration % PPH not described	Csapo microballoon with Statham P-1000A pressure transducer Duration of monitoring: 20 min Timing with regard to placental expulsion: not described	Basal tone, frequency, intensity and duration of UC. UA in MVU per 10 min. UA analysis method: not described	25 mg ketamine iv has no effect on postpartum UA. 50, 75 and 100 mg increases intensity and frequency of UC
Baumgarten, 1983, Austria (42)	Multicenter RCT	Effect of different uterotonics on postpartum UA	75	*Inclusion:* Low-risk, term pregnancy (38–41 weeks of gestation) Spontaneous delivery No oxytocin during first stage of labor *Exclusion:* Long duration of labor (>12 h) *Postpartum medication:* Timing: 30 min after IUPC placement Methergin 200 μg im or syntocinon 2 IU im or sulprostone 500 μg im or saline solution im % PPH not described	Twin-catheter (unknown) First 210 min after placental expulsion Inserted immediately after placental expulsion	Average frequency and intensity UA calculated per 30 min in MVU	Sulprostone’ s effect on UA is superior to methergin and oxytocin, particularly in terms of rapid onset and intensity. However, methergin has the most prolonged effect on UC
Endl, 1981, Germany (43)	Double-blind prospective study	Effect of various sort and dosis B-mimetica on postpartum UA	Unknown	*Inclusion:* Oxytocine during labor *Exclusion:* not described *Postpartum medication:* Standard oxytocin infusion 20 mU/min 30 min after IUPC placement: ○ Oral medication (5 mg fenoterol or 10 mg fenoterol or 0.5 mg hexoprenalin or 10 mg ritodine or 20 mg ritodrine) or placebo 180 min after IUPC placement: 1 ampul (unknown dose) methergin iv, subsequently discontinuing UA monitoring % PPH not described	Type of catheter not described First 180 min after placental expulsion; measuring UA after 30, 60, 120 and 180 min, respectively Inserted immediately after placental expulsion	% change of UA (30 min pre-treatment vs. 30, 60, 120 and 180 min post-treatment) UA analysis method: not described	Standard dosages of oral B-mimetics may not be effective for long-term treatment of threatened preterm labor postpartum, as only a double dose of fenoterol showed sufficient efficacy after120 and 180 min
Forman, 1982, Sweden (44)	Prospective observational study	Effects of nifedipine on spontaneous and methylergometrine-induced UA postpartum	17	*Inclusion:* Low-risk pregnancy and delivery VD No medical treatment during labor *Exclusion:* not described *Postpartum medication:* Nifedipine 30 mg po, 30 min after IUPC placement Methylergometrine 0.15 mg po, 20 min after IUPC placement Methylergometrine 0.15 mg po, 20 min after IUPC placement + nifedipine 30 mg po 25–40 min after methylergometrine % PPH not described	Millar instruments First 60–240 min after placental expulsion Inserted immediately after placental expulsion	Calculated UA in MVU per 10 min	Nifedipine can effectively reduce spontaneous and methylergometrine-induced UA postpartum
Forman, 1982, Sweden (45)	Prospective observational study	Effects of nifedipine on oxytocin-induced UA and PGF_2a_ induced UA postpartum	19	*Inclusion:* Low-risk, a term pregnancy and delivery VD No medical treatment during labor *Exclusion:* not described *Postpartum medication:* Oxytocin iv 20mIU/min, 20–30 min after IUPC placement Oxytocine iv mIU/min 20–30 min after IUPC placement, Nifedipine 30 mg po 25–40 min after oxytocine PGF_2a_ iv 35 μg/min 20–30 min after IUPC placement PGF_2a_ iv 35 μg/min 20–30 min after IUPC placement + nifedipine 30 mg po 20–50 min after PGF_2a_ % PPH not described	Millar instruments First 110–150 min after placental expulsion Inserted immediately after placental expulsion	Calculated UA in MVU per 10 min	Nifedipine can effectively reduce oxytocin-induced and PGF_2a_-induced UA postpartum
Egarter, 1988, Austria (46)	Prospective cohort study	Effect of B-blocker Pindolol on postpartum UA	16	*Inclusion:* Low-risk VD Singleton pregnancy, cephalic position *Exclusion*: not described *Postpartum medication*: Routine use of 5 IE syntocinon (unknown route of administration) postpartum, no other uterotonics Single dose: 0.8 mg Pindolol iv 10 min after IUPC placement Double dose: 0.8 mg Pindolol iv 10 and 0.8 mg Pindolol iv 50 min after IUPC placement % PPH not described	Hewlett Packard 14 09 90 (Intran^®^ Plus) First 60 – 110 min after placental expulsion Inserted immediately after placental expulsion	Average basal tone, frequency and intensity Calculated UA in MVU per 10 min	B-blocker Pindolol (0.8 and 1.6 mg iv) reduced UA, without side effects
Egarter, 1989, Austria (47)	RCT, double-blind	Effect of B-blocker Pindolol on postpartum UA	22	*Inclusion:* Low-risk VD No oxytocin or prostaglandins during labor *Exclusion*: no medication or previous analgesics or anesthetics during labor *Postpartum medication*: Single dose: 0.8 mg Pindolol iv 10 min after IUPC placement Double dose: 0.8 mg Pindolol iv 10 and 0.8 mg Pindolol iv 60 min after IUPC placement % PPH not described	Hewlett Packard 14 09 90 (Intran^®^ Plus) First 60 – 110 min after placental expulsion Inserted immediately after placental expulsion	Average basal tone, frequency and intensity Calculated UA in MVU per 10 min	B-blocker Pindolol (0.8 and 1.6 mg iv) reduced UA, without side effects
Granström, 1989, Sweden (64)	Prospective cohort study	Effect of 15 methyl-prostaglandin F_2a_ infusion on postpartum UA in PPH population	5	*Inclusion:* AD 36–42 weeks of gestation VD Heavy bleeding and signs of uterine atony after failed therapy of oxytocin and methergin *Exclusion*: partially retained placenta *Postpartum medication:* Oxytocin 10 IE im, directly postpartum Methergin 0.2 mg iv and additional oxytocin 40 IE iv in case of heavy bleeding and uterine atony 15-methyl prostaglandin F_2a_ iv infusion in case of failed therapy of methergin and oxytocin (started at 20 drips/min), increased every 10 min until a max of 40 drips/min. *Postpartum blood loss*: not described 100% PPH (mean blood loss 1,500 mL, range 700–2,900 mL)	Gaeltec catheter Total of 70 min postpartum (10 min before 15-methyl-prostaglandin F_2a_ infusion until 60 min after start infusion) Timing with regard to placental expulsion not described	Amplitude (in mmHg) and frequency of UC UA analysis method: not described	15-methyl prostaglandin F_2a_ infusion by PPH population resulted in high amplitude and frequency of UC, cessation of bleeding within 12.5 min (mean)
Ingemarsson, 1989, Sweden (57)	Prospective cohort study	Effect of Isradipine iv on postpartum UA and TBL	34	*Inclusion:* VD, cephalic position Spontaneous UA for Isradipine 0.5 mg iv and control group, oxytocin infusion during labor and postpartum for Isradipine 1 mg iv and control group *Exclusion*: not described *Postpartum medication:* Isradipine 0.5 mg iv in 5 min, 30 min after IUPC placement and control group Oxytocin 12.5 mU/min iv during labor and postpartum + isradipine 1 mg iv. 30 min after IUPC placement and control group Oxytocin 12.5 mU/min iv during labor and postpartum + terbutalin 0.25 mg iv in 5 min, 35 min after IUPC placement *Postpartum blood loss:* weighted 2.9% PPH	Gaeltec catheter During 150 min after childbirth Inserted 30–45 min after childbirth	% increase in calculated UA in MVU (30 min pre-treatment vs. 120 min post-treatment)	Isradipine given as bolus injection can reduce pp. UA with minimal side effects
Chua, 1993, Singapore (48)	Prospective study	Effect of methergin and syntocinon stored at different high temperatures on postpartum UA	Unknown	*Inclusion:* Low-risk, spontaneous labor, normal VD No antenatal or intrapartum complications, interventions or medication *Exclusion:* not described *Postpartum medication:* Timing: 30 min after IUPC placement Syntocinon 5 IE iv or methylergometrine 0.2 mg iv stored at 22, 32, 37 or 42 °C for, respectively, 3,6,9 and 12 months % PPH not described	Gaeltec catheter First 60 min after placental expulsion Inserted immediately after placental expulsion	% increase in UA (30 min pre-treatment vs. 30 min post-treatment) UA analysis method: not described	Both uterotonics increased UA, but their efficacy decreased when stored at higher temperatures
Chua, 1994, Singapore (49)	Prospective cohort study	Effect of breastfeeding and nipple stimulation on postpartum UA	11	*Inclusion:* Spontaneous labor, term pregnancy, normal VD *Exclusion*: intervention or medication during labor *Postpartum medication:* none *Procedure:* nipple stimulation or breastfeeding 30 min after IUPC placement % PPH not described	Gaeltec catheter First 60 min after placental expulsion (30-min post-stimulation compared to 30-min pre-stimulation) Inserted immediately after placental expulsion	Active pressure in mmHg for each contraction, calculated of the manual recorded peak pressure minus the average basal tone. Calculated cumulative UA in MVU per 15-min	Significant increase in postpartum UA after breastfeeding or nipple stimulation
Chua, 1996, Singapore (50)	Prospective cohort study	Description of relationship between postpartum UA an TBL	27	*Inclusion:* Spontaneous labor, normal VD *Exclusion*: not described *Postpartum medication:* 17/27 active postpartum management with oxytocics and CCT Syntometrine im (unknown dose) or PGF_2a_ im (Carboprost^®^) im (unknown dose) at delivery of the anterior shoulder *Postpartum blood loss*: measured by colorimetric measurements of hemoglobin 3.7% PPH	Gaeltec catheter First 120 min after placenta expulsion Inserted <5 min after placental expulsion	Cumulative active pressure in mmHg, manually calculated by summation of the amplitudes observed every 30s from the contraction curve	Poor correlation between UA and TBV after placental birth
Choo, 1998, Singapore (65)	Prospective cohort study	Description of relationship between postpartum UA an TBL after administration of different uterotonics	47	*Inclusion:* Low-risk, singleton pregnancy Spontaneous labor *Exclusion:* Grand multipara, prolonged labor, history of PPH or antepartum hemorrhage in current pregnancy *Postpartum medication* Timing: 30–45 min after IUPC placement Syntocinon iv (unknown dose) or syntometrine im (unknown dose) or oral misoprostol: 200, 400, 500 or 600 μg) *Postpartum blood loss:* visual assessment 15% PPH	Gaeltec catheter First 120 min after placental expulsion Inserted <5 min after placental expulsion	Cumulative UA in mmHg per 15 min Manually calculated on the amplitudes observed every 30s from the contraction curve	Poor correlation between postpartum UA and TBL after placental expulsion and administration of uterotonics
Amsalem, 2014, Canada (52)	Prospective cohort study	Effect of low-dose im carbetocin compared to high-dose im oxytocin on postpartum UA	12	*Inclusion:* Low-risk, term (37–40 weeks), singleton pregnancy Spontaneous labor, normal VD No oxytocin during labor Epidural analgesia *Exclusion:* Grand multipara (parity >4) *Postpartum medication:* Timing: direct after IUPC placement Oxytocin 10 IU im (high-dose) or carbetocin 30 μg im (low-dose) 8% PPH	Millar Instruments First 90 to 360 min after placental expulsion Inserted immediately after placental expulsion	Mean maximum amplitude (change from baseline in mmHg), frequency of contractions, mean duration of contractions (seconds) UA calculated from the tocographic data (without artifacts) per 10 min in MVU, visual interpretation	Low-dose carbetocin im had a higher amplitude and frequency of contractions compared to high-dose oxytocin im
Chong, 2001, Singapore (53)	Prospective descriptive study	Effect of different dosages of oral misoprostol compared to im syntometrine on postpartum UA and side effects	57	*Inclusion:* Singleton pregnancy Spontaneous labor, normal VD No oxytocin or prostaglandins during labor *Exclusion:* Anemia, history of PPH or antepartum hemorrhage in current pregnancy *Postpartum medication:* Active postpartum management with oxytocics Syntometrine im 1 mL (oxytocin 5 units, ergometrine maleate 500 μg/mL) or oral misoprostol: 200, 400, 600 or 800 μg, 30 min after placental expulsion % PPH not described	Gaeltec catheter First 120 min after placental expulsion Inserted <5 min after placental expulsion	Automatic detection and calculation of mean cumulative UA in kPas/s (90 min post-treatment vs. 30 min pre-treatment)	Uterotonic effects of all five dosages of oral misoprostol was not statistically significant to that of syntometrine im. Less side effects were seen ≤400 μg oral misoprostol
Chong, 2004, Singapore (54)	Prospective cohort study	Effect of different routes of misoprostol 400 μg compared to syntometrine im on postpartum UA and side effects	50	*Inclusion:* Singleton pregnancy Spontaneous labor, normal VD No oxytocin or prostaglandins during labor No epidural analgesia *Exclusion:* Anemia, history of PPH or antepartum hemorrhage in current pregnancy, maternal infection *Postpartum medication:* Active postpartum management with oxytocics Syntometrine im 1 mL (oxytocin 5 units, ergometrine maleate 500 μg/mL), 30 min after placental expulsion Misoprostol 400 μg oral tablet, oral solution, rectal or vaginal, 30 min after placental expulsion 0% PPH	Gaeltec catheter First 120 min after placenta expulsion Inserted <5 min after placental expulsion	Cumulative UA per 15-min in kPas/s (90 min post-treatment vs. 30 min pre-treatment, taking parity and pre-treatment cumulative UA were taken into account) Automatic detection of UA and visual inspection of onset of action (blinded for treatment)	Oral solution misoprostol had the fastest and highest uterotonic effect, but also the most side effects
Schorn, 2009, USA (59)	RCT	Pilot study to test the effects of guided imagery on blood loss during the third stage of labor	41	*Inclusion:* 36–38 weeks of gestation, singleton pregnancy Spontaneous VD *Exclusion*: Grand multipara (>4), history of PPH, bleeding disorder, seizure disorder, polyhydramnios, (gestational) diabetes, hypertension, cardiac disease, uterine fibroids, anemia, intrauterine fetal demise or tobacco use *Postpartum medication*: oxytocin postpartum, conform local protocol. *Postpartum blood loss:* weighted 4.9% severe PPH (≥1,000 mL), % PPH ≥ 500 mL not described	Covidien Kendall Accu-Trace catheter Inserted immediately after childbirth until expulsion of IUPC due to placental expulsion	Contraction frequency and intensity in mmHg UA analysis method: not described	No significant differences between the 3 groups on the contraction frequency, contraction strength and total blood loss
Elati, 2011, United Kingdom (55)	Single center, randomized trial combined with results of a previous pilot study	Comparing postpartum UA and the effect of three doses sublingual misoprostol with oxytocin im	49	*Inclusion:* Singleton pregnancy Spontaneous labor, normal VD No oxytocin during labor *Exclusion:* Anemia, history of PPH or antepartum hemorrhage in current pregnancy, maternal infection, previous CD, polyhydramnios *Postpartum medication:* Active postpartum management with uterotonics and CCT Sublingual misoprostol (200, 400 or 600ug) or oxytocine 10 IU im (previous pilot study) ≤ 1 min after childbirth % PPH not described	Koala External Balloon Catheter IPC-5000E First 120 min after placental expulsion Inserted immediately after placental expulsion	Calculated mean UA in MVU per 10 min UA (in MVU) is adjusted for the first 10 min after delivery	Oxytocin im has the highest immediate postpartum UA response Misoprostol at lower doses was found to be just as effective as at higher levels, with less adverse effects
Schorn, 2012, USA (58)	Prospective study	Evaluate the use of IUPC to measure UA following the birth through expulsion of the placenta	36	*Inclusion:* Low-risk, singleton pregnancy 36–38 weeks of gestation Normal VD *Exclusion:* Grand multipara (>4), history of PPH, bleeding disorder, seizure disorder, polyhydramnios, (gestational) diabetes, hypertension, cardiac disease, uterine fibroids, anemia, intrauterine fetal demise or tobacco use *Postpartum medication:* 18/36 active postpartum management with oxytocin conform local protocol % PPH not described	Covidien Kendall Accu-Trace catheter Inserted immediately after childbirth until expulsion of IUPC due to placental expulsion	Contraction frequency and intensity in mmHg UA analysis method: not described	52% of participants had identifiable contraction patterns
Electrophysiological monitoring							
Rottinghuis, 1962, Germany (61)	Prospective cohort	Feasibility of UA pp. monitoring	Unknown	*Inclusion*: not described *Exclusion*: not described *Postpartum medication*: not described % PPH not described	EHG: detailed characteristics not described Duration of monitoring: minimal 30 min postpartum	Frequency, amplitude, duration, intensity in μV UA analysis method: not described	Postpartum EHG monitoring is feasible
Rosen, 2014, Israel (60)	Prospective observational mono-center study	Comparison of UA in the third stage of labor with UA in the second stage of labor	44	*Inclusion:* Spontaneous, term singleton pregnancy VD *Exclusion*: not described *Postpartum medication*: none % PPH not described	EHG: EUM-100, 9 electrodes and 1 ground electrode on the left thigh. Timing: final 30 min second stage of labor until placental expulsion Third stage of labor: replacement of abdominal electrodes was possible when deemed necessary by the researcher (exact position not described)	Mean electrical activity of uterine muscle per 10 min (micro-Joule) Automatic data analyzer (blinded to clinical outcome)	Similar UA (and intensity) of contractions in the third stage of labor compared to the final 30 min of the second stage of labor
Mas-Cabo, 2020, Spain (36)	Use of retrospective database of multicenter study	Assess the performance of EHG parameters postpartum	8	*Inclusion:* Term pregnancy Spontaneous, vaginal delivery *Exclusion*: not described *Postpartum medication*: not described % PPH not described	EHG: 2 disposable monopolar Ag/AgCl electrodes with interelectrode distance of 8 cm. Placed under umbilicus (lower compared to position of electrodes during labor). Data acquisition device: not described. Timing: ± 30–60 min during the first 3 h after delivery	Temporal and spectral parameters: root mean square (0.1–4 Hz), dominant frequency (0.34–1 Hz), decile 3, 5 and 7 of the power spectrum, spectral moment ratio Non-linear parameters (0.34–4 Hz): Lempel-Ziv complexity (binary form), sample entropy, time reversibility, spectral entropy, ratio SD1/SD2 Visual removal of artifacts, computed whole-window analysis of UA (moving windows of 120 s with 50% overlap)	Postpartum physiological changes in the uterus are characterized by a decrease in amplitude-related and spectral parameters, including deciles and dominant frequency, indicating reduced uterine cell excitability. Conversely, the spectral moment ratio, Lempel–Ziv complexity, sample entropy, spectral entropy, and SD1/SD2 values increased postpartum
Diaz-Martinez, 2020, Spain (38)	Prospective observational mono-center study	Feasibility of postpartum EHG monitoring Comparing postpartum EHG characteristics of vaginal and elective cesarean deliveries	33	*Inclusion:* Low-risk, term, singleton pregnancy VD or elective CD No oxytocin during labor *Exclusion*: Fetal macrosomia, polyhydramnios, maternal age > 45 years *Postpartum medication*: Active postpartum management with oxytocics: VD: oxytocin 20 IE iv conform local protocol CD: Carbetocin 100 μg iv conform local protocol % PPH not described	EHG: 4 Ag/AgCl electrodes (interelectrode distance 3 cm) positioned on uterine fundus under umbilicus with 2 reference electrodes on each hip. Data acquisition device: custom-made Timing: ± 60 min during the first 3 h after childbirth and placental expulsion	Spectral, temporal, and non-linear parameters (0.1–4 Hz): peak-to-peak amplitude, kurtosis of Hilbert envelope, median and dominant frequency, normalized energy, sample entropy, spectral entropy, Lempel-Ziv binary complexity, Katz fractal dimension Visual removal of artifacts, computed whole-window analysis of UA (moving windows of 120 s with 50% overlap)	UA is more frequent and intense (higher amplitude and spectral content shift to higher frequencies) after VD compared to elective CD
Gray, 2020, USA (37)	Prospective observational study	Relationship between simultaneous recording of uterine, abdominal and pelvic floor muscles during the second and third stages of labor	28	*Inclusion:* Term pregnancy Spontaneous delivery *Exclusion:* not described *Postpartum medication:* not described % PHH not described	EHG: 4 electrodes of maternal abdomen around umbilicus. Timing: directly after childbirth until placental expulsion	Total power, peak PDS, root mean square, burst duration Automatic detection and calculation of UA PowerLab hardware and LabChart software (ADinstruments)	Electrical activity of the uterine, pelvic and abdominal muscles significantly increased postpartum compared to the second stage of labor.
Paljk Likar, 2022, Slovenia (39)	Single-center randomized, open-label trial	Comparing the postpartum EHG characteristics of elective cesarean deliveries after carbetocin im or oxytocin iv	57	*Inclusion:* Singleton, term pregnancy, elective CD because of CD in history *Exclusion:* Contra-indication for oxytocin or carbetocin, anemia, history of PPH, uterine fibroids, blood clotting disorder, placenta praevia, placenta accrete, hypertensive disorders of pregnancy, renal-, cardiac- or hepatic dysfunction *Postpartum medication:* Oxytocin 5 IU iv or Carbetocin 100 μg im after ± 15 min EHG recording 6/57 received ergometrine due to increase blood loss or inadequate uterine contractility % PPH not described	EHG: two sets of bipolar electrodes placed three to 4 cm around the navel, using custom-built uterine EMG patient-monitor system (SureCall Monitor) Directly at the high dependency perinatal unit, after CD Timing: unknown with regard to time after CD. Total duration of monitoring 75 min	Various linear EHG parameters: PDS peak frequency (Hz), PDS peak amplitude (Hz), interval between pseudo-bursts (s), pseudo-burst duration (s), number of pseudo-bursts, PDS integral (μV). Computerized data analyzer with Labchart 8 software (ADInstruments). Movements and noise were noted and excluded from analysis. 0.3–1 Hz frequencies, using Fourier transform and Cosine-bell windowing.	Significant higher PDS peak frequency 2 h after uterotonics in oxytocin group vs. carbetocin group
Thijssen, 2023, The Netherlands (40)	Prospective observational multi-center study	Exploring the potential of EHG to detect postpartum UA + patient satisfaction survey of EHG monitoring postpartum	91	*Inclusion:* High-risk (CD in history and/or BMI ≥ 30 and/or induced labor and/or inadequate TOCO recording during labor) VD *Exclusion:* water birth, skin diseases or allergies, external or implanted electrical stimulator *Postpartum medication:* conform local protocol (± 90% postpartum oxytocin) *postpartum blood loss*: not described 10% PPH defined as >1,000 mL/24 h after childbirth	EHG: 3 electrodes. Skin Impedance < 5 *Ω*. Reference electrode not described. Graphium and PUREtrace (Nemo Healthcare BV) 2 different patch positions: next to or under umbilicus Timing: 1 h before start pushing until > 1 h after childbirth	Mean contraction frequency per hour Manually removal of artifacts, visual readability of UA by researchers Contraction annotation software tool	It is possible to detect UA postpartum with EHG 73% of women experienced no inconvenience from postpartum EHG monitoring
Frenken, 2025, The Netherlands (41)	Prospective explorative mono-center study	Associations between EHG parameters in the first 30 min postpartum and TBL, subgroups PPH vs. no PPH.	25	*Inclusion:* Singleton pregnancy 36–42 weeks of gestation VD *Exclusion:* water birth, maternal abdominal dermatologic diseases, fetal cardiac arrhythmias, external or implanted electrical stimulators *Postpartum medication:* Active postpartum management with 5 IU oxytocin iv directly after childbirth Additional medication (i.e., methylergonovine and tranexamic acid) could be administered *Postpartum blood loss*: weighted 12.0% PPH	EHG: 4 electrodes (interelectrode distance: not described) with 2 reference electrodes on the abdomen. Skin impedance <5 kΩ Nemo Fetal Monitoring System Timing: first 30 min after childbirth	AUC total, AUC from baseline, maximum amplitude, baseline tone (all in arbitrary units) from EHG-derived tocogram Visual removal of artifacts, computed whole-window analysis of UA with UA annotator	Moderate positive correlation between TBL and postpartum EHG parameters. All parameters were significantly higher in women with PPH.
TOCO							
Masuzawa, 2017, Japan (56)	Prospective multicenter observational study	Describe postpartum UA after placental expulsion in low risk women monitored with TOCO	17	*Inclusion:* Low-risk, singleton, term pregnancy No medical treatment during labor *Exclusion:* emergency PPH treatment *Postpartum medication:* 2/17 oxytocin 5 IU iv *Postpartum blood loss:* weighted 5.8% PPH	Fetal Actocardiograph MT-235, MT-516 First 120 min after placental expulsion	Mean contraction parameters (interval (sec), intensity (mmHg) and duration (sec)) per 10 min Visual interpretation and calculated	No correlation was found between UC frequency and TBL

CCT, Cord controlled traction; CD, cesarean delivery; EHG, electrohysterography; EMG, electromyography; IE/IU, international units; iv, intravenous; im, intramuscular; IUPC, intrauterine pressure catheter; po, per os; mg, milligram; min, minutes; mU, milli Units; MVU, Montevideo Units; PPH, postpartum hemorrhage; RCT, Randomized Controlled Trial; Sec, seconds; TBL, total blood loss; VD, vaginal delivery; Vol, volume; UA, uterine activity; UC, uterine contraction.

**Figure 2:**
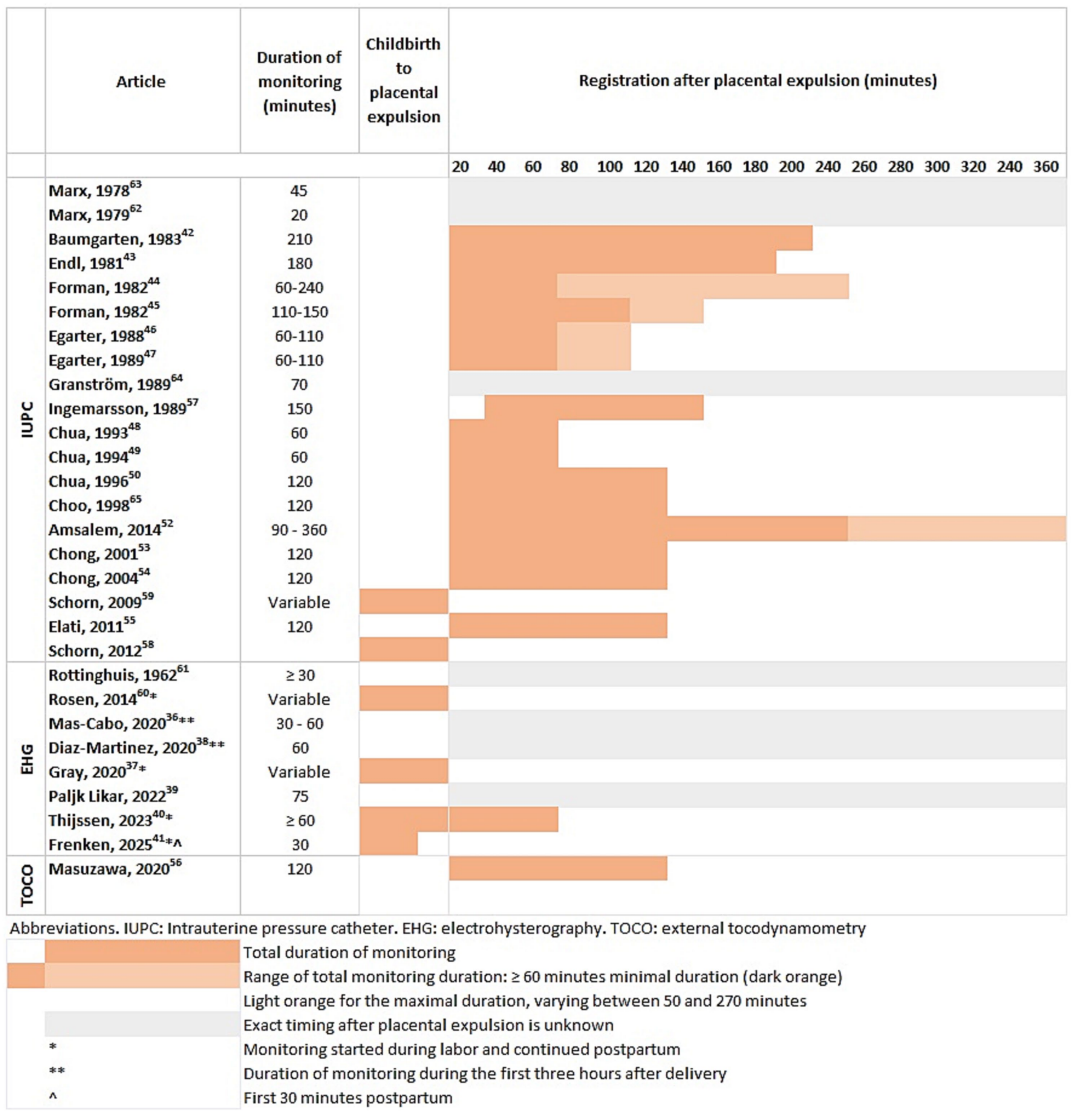
Overview of the timing and duration of postpartum monitoring.

The most commonly studied postpartum monitoring technique was IUPC (n = 20), followed by EHG (n = 8) and TOCO (n = 1). Since 2020, only articles with EHG as postpartum monitoring method have been published (36–41). The timing and duration of postpartum monitoring varied in the included studies and were categorized in the third stage of labor (childbirth to placental expulsion), the period after childbirth and the period after placental expulsion (Figure 2).

In 15 out of 29 articles, UA was directly measured after placental expulsion with measurements lasting 20 to 360 min (42–56). One study started monitoring 30–45 min after placental expulsion for a total of 150 min (57). Four studies only monitored UA during the third stage of labor (37, 58–60), one study monitored UA from childbirth to 30 min postpartum (41), and one study from childbirth until ≥ 1 h postpartum (40). Furthermore, seven studies monitored UA for 20–75 min after childbirth. However, the exact duration was unclear (36, 38, 39, 61–64).

The included articles can be categorized into five overarching topics, although some studies may fall under more than one category: (1) the effect of various sort, routes and dosages of medication on postpartum UA (n = 17) (39, 42–48, 52–55, 57, 62–65), (2) the feasibility of postpartum monitoring with different monitoring methods (n = 14) (36–38, 40, 41, 44, 45, 50, 52, 57–59, 61, 65), (3) postpartum UA in relation with postpartum blood loss (n = 8) (40, 41, 50, 56, 57, 59, 64, 65), (4) mode of delivery and the relation with postpartum UA (n = 1) (38), and (5) UA during the stages of labor (n = 3) (37, 40, 60).

### Risk of bias of included studies

**Figure 3:**
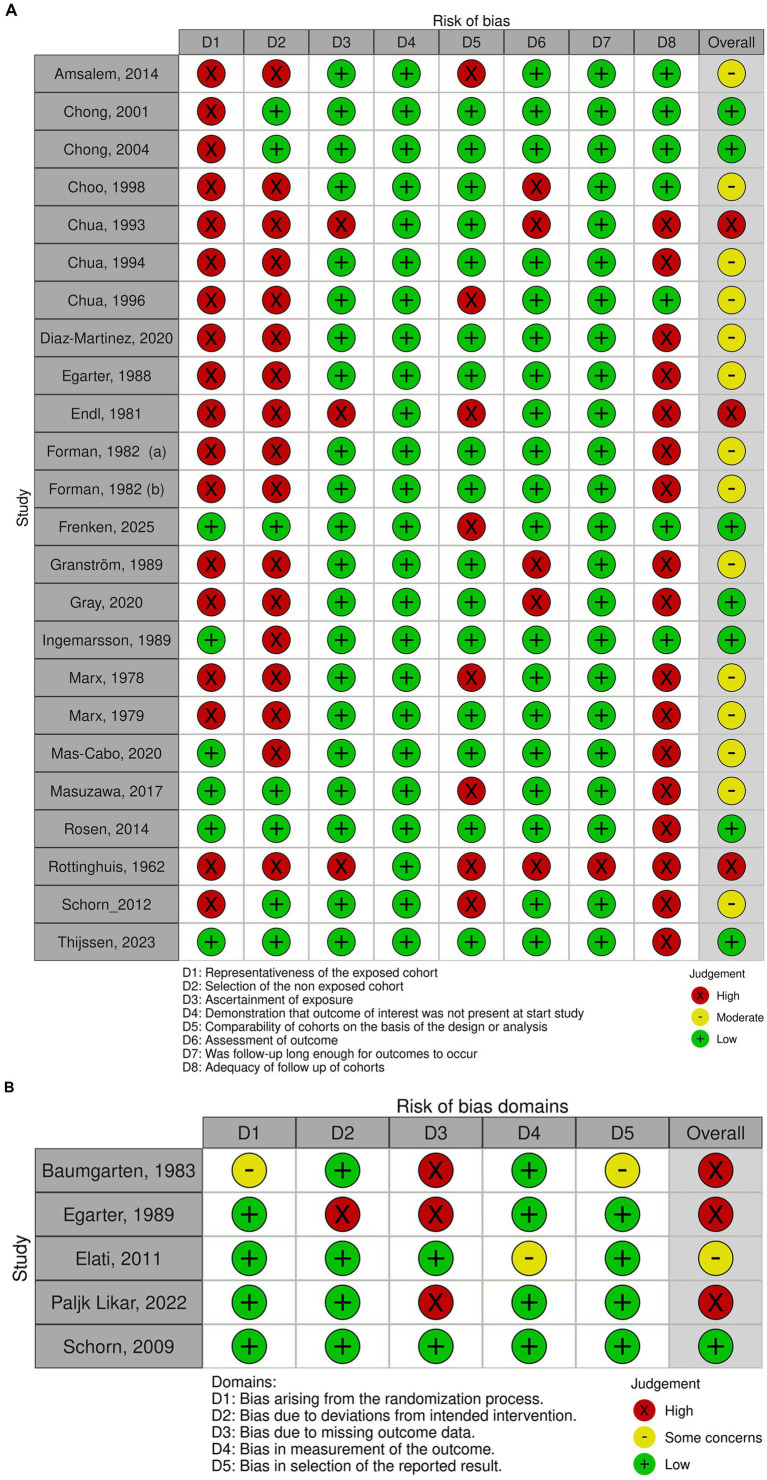
Quality of bias evaluation of included studies according to NOS and RoB 2 conducted with Risk-of-bias VISualization (robvis) tool (70). **(A)** NOS for cohort studies. **(B)** RoB 2.0 for RCTs.

An overview of the risk of bias assessment of the included studies is presented in Figure 3. In total, six studies were excluded from data synthesis because of high risk of bias (IUPC: n = 4 and EHG: n = 2) (39, 42, 43, 47, 48, 61).

### What is known about the physiology of postpartum UA?

Seventeen studies outlined postpartum UA (36–38, 40, 41, 44, 45, 49, 52–54, 56–60, 62). Table 2 shows the results of postpartum UA characteristics per UA parameter. Supplementary File 3 describes the postpartum UA characteristics per postpartum stage.

**Table 2:** Results of postpartum uterine activity divided by: (A) uterine contraction interval, (B) uterine contraction frequency per 10 min, (C) uterine contraction duration in seconds, (D) baseline uterine activity, (E) uterine intensity (i.e., maximum amplitude) and (F) cumulative uterine activity.

(A). Uterine contraction interval						
First author, year	*N*	Monitoring method	Profylactic oxytocin postpartum	Timing	Definition and unit	Result: mean ± SD (range lowest−highest value)
Schorn, 2009 (59)	41	IUPC	Conform local protocol	Third stage	Onset-to-onset time between contractions in minutes	2.75 ± 0.29 (1–5)
Schorn, 2012 (58)	36	IUPC	18/36 participants received oxytocin conform local protocol	Third stage	Onset-to-onset time between contractions in minutes	2.6 ± 0.9 (1–5)
Masuzawa, 2017 (56)	17	TOCO	2/17 participants received 5 IU oxytocin IV	Third stage	Minutes between the first contraction peak to the second contraction peak	2.4 ± 0.9
				First 60 min after placental expulsion		4.2 ± 0.7
				60–120 min after placental expulsion		7.9 ± 2.1

(B). Uterine contraction frequency per 10 min					
First author, year	*N*	Monitoring method	Profylactic oxytocin postpartum	Timing	Result: mean ± SD (range lowest–highest value)
Marx, 1979^a^ (62)	22	IUPC	None	First 10 min after placental expulsion	(2.4 ± 0.2–2.8 ± 0.5)
Forman, 1982 (44)	17	IUPC	None	First 10 min after placental expulsion	2.6
Egarter, 1988 (46)	16	IUPC	None	First 10 min after placental expulsion	2.6 ± 0.6
Ingemarsson, 1989^b^ (57)	34	IUPC	None	Duration of 30 min, 30–45 after childbirth	2.7
				Duration of 15 min, 45–60 min after childbirth	2.4
			12.5 mU/min IV	Duration of 30 min, 30–45 after childbirth	3.7
				Duration of 120 min, 60–75 min after childbirth	2.1
Amsalem, 2014 (52)	12	IUPC	10 IU oxytocin IM	First 60 min after placental expulsion	4.6
				60–240 min after placental expulsion	2.1
Thijssen, 2023^c^ (40)	91	EHG	90% received oxytocin conform local protocol	Third stage	3.7
				60 min postpartum	2.7
				120 min postpartum	2.2

(C). Uterine contraction duration in seconds						
First author, year	*N*	Monitoring method	Profylactic oxytocin postpartum	Definition & unit	Timing	Result: mean ± SD
Masuzawa, 2017 (56)	17	TOCO	2/17 participants received 5 IU oxytocin IV	Duration in seconds at the point of one-fifth intensity from baseline	Third stage	54.4 ± 20.9
					First 60 min after placental expulsion	83.3 ± 11.2
					60–120 min after placental expulsion	102.7 ± 25.9

(D). Baseline uterine activity^e^							
First author, year	*N*	Monitoring method	Profylactic oxytocin postpartum	Timing	Definition	Unit	Result: mean ± SD or median with [interquartile range] (range lowest–highest value)
Marx, 1979^a^^,^ ^d^ (62)	22	IUPC	None	Unknown time period after placental expulsion, duration 10 min	No definition for resting pressure	mmHg	(22.5 ± 2.8–26.7 ± 4.0)
Forman, 1982 (44)	17	IUPC	None	First 10 min after childbirth	No definition for basic tone	mmHg	<10
Forman, 1982 (45)	19	IUPC	None	First 20–30 min after childbirth	No definition for basic tone	mmHg	10
Egarter, 1988 (46)	16	IUPC	None	First 10 min after childbirth	No definition for basal tone	mmHg	7.9 ± 3.4
Ingemarsson, 1989 (57)	34	IUPC	None	Duration of 30 min, 30–45 min after childbirth	No definition for basal tone	mmHg	10–15
Frenken, 2025 (41)	23	EHG	5 IU oxytocin IV	First 30 min after childbirth	Baseline: 10th percentile of all UA in 30 min	Arbitrary units	12.1 [8.1–23.1]

(E). Uterine intensity (i.e., maximum amplitude)							
First author, year	*N*	Monitoring method	Profylactic oxytocin postpartum	Timing	Range	Unit	Result: mean ± SD or median with [interquartile range] (range lowest–highest value)
Marx, 1979^a^ (62)	22	IUPC	None	First 10 min after placental expulsion	0–260	mmHg	(93.3 ± 15.4–110.0 ± 10.7)
Forman, 1982 (44)	17	IUPC	None	First 10 min after childbirth	110–350	mmHg	160
Egarter, 1988 (46)	16	IUPC	None	First 10 min after childbirth	Not described	mmHg	69.1 ± 26.7
Schorn, 2009 (59)	41	IUPC	Conform local protocol	Third stage	20–100	mmHg	51.7 ± 26.2
Schorn, 2012 (58)	36	IUPC	18/36 participants received oxytocin conform local protocol	Third stage	20–100	mmHg	58.0 ± 30.7
Masuzawa, 2017 (56)	17	TOCO	2/17 participants received 5 IU oxytocin IV	Third stage	Not described	mmHg	56.1 ± 13.3
				First 60 min after placental expulsion	Not described	mmHg	50.8 ± 18.1
				60–120 min after placental expulsion	Not described	mmHg	50.8 ± 18.1
Gray, 2020 (37)	28	EHG	Not described	Third stage	Not described	V^2^E^−8^	7
Frenken, 2025 (41)	23	EHG	5 IU oxytocin IV	First 30 min after childbirth	Not described	Arbitrary units	303.0 [176.4–761.1]

(F). Cumulative uterine activity							
First author, year	*N*	Monitoring method	Profylactic oxytocin postpartum	Timing	Definition	Unit	Result: mean ± SD or median with [interquartile range] (range lowest–highest value)
Forman, 1982^e^ (44)	17	IUPC	None	First 10 min after childbirth	Not described	MVU	420
Forman, 1982^e^ (45)	19	IUPC	None	First 45–70 min after placental expulsion	Not described	MVU	395 ± 38.2
Egarter, 1988 (46)	16	IUPC	None	First 10 min after childbirth	Not described	MVU	176.6 ± 76.5
Chua, 1994 (49)	11	IUPC	None	30–60 min after placental expulsion	Average UA of two 15-min values. Active pressure per contraction: manual recording of the peak pressure minus the average basal tone in a 15 min window.	MVU	(5–1,313), mean value 612 MVU per 15 min, i.e., 408 per 10 min
Chong, 2001 (53)	57	IUPC	Conform local protocol	First 30 min after placental expulsion	Baseline cumulative uterine activity over 30 min	kPA*s	(5,358 ± 1877–7216 ± 2,776)
Chong, 2004 (54)	50	IUPC	Conform local protocol	First 30 min after placental expulsion	Baseline cumulative uterine activity over 30 min	kPA*s	3772.5 ± 3320.7–5806.0 ± 3610.5
Rosen, 2014 (60)	44	EHG	None	Third stage	Not described	mW*s	3.4 ± 0.6
Frenken, 2025 (41)	23	EHG	5 IU oxytocin IV	First 30 min after childbirth	Total area under the contraction curve	Arbitrary units	96486.1 [62394.5–139526.6]

EHG, electrohysterography; IM, intramuscular; IU, international units; IUPC, intrauterine pressure catheter; IV, intravenous; KPA*s, kilopascal-seconds; Min, minutes; mmHg, millimeters of mercury; mU, milli-Units; MVU, Montevideo Units; mW*s, milliwatt-seconds; SD, standard deviation; TOCO, external tocodynamometry; UA, uterine activity; V, velocity.

a All participants have an pundendal block with or without nitrous oxide-oxygen analgesia or with nitrous oxide-oxygen analgesia alone.

b Uterine contraction frequency is calculated from 15 to 10 min.

c Uterine contraction frequency is calculated from 60 to 10 min.

d Resting pressure is calculated from Torr to mmHg.

e Maximum intensity of amplitude is not described, only 350 mmHg in both studies of Forman et al.

#### Uterine contraction interval, frequency and duration

Three studies reported the mean uterine contraction interval (UCI) during the postpartum period (Table 2A) (56, 58, 59). UCI was defined as the time between the uterine contractions in min, and the reported ranges during the third stage of labor was between 2.4 ± 0.9 and 2.8 ± 0.9 min. Furthermore, six studies reported postpartum UCF/10 min (Table 2B) (44, 52, 57, 62). In the study of Thijssen et al. EHG was used to measure UCF during the third stage of labor. They reported a median UCF of 3.7/10 min. In this study almost all participants received prophylactic oxytocin conform local protocol (66). In three studies, prophylactic oxytocin administration was not used. These studies reported a range of the mean UCF between 2.4 and 2.8/10 min in the first 10 min after placental expulsion (44, 46, 62). A comparable mean UCF of 2.7/10 min, without prophylactic oxytocin administration, was described 30–45 min after childbirth (57). Besides, several studies demonstrated an increase in UCI and reduction in UCF over time, monitored with either IUPC, EHG and TOCO (40, 44, 52, 56, 57). However, with the administration of prophylactic oxytocin, higher mean UCF/10 min of 3.7, 30–45 min after childbirth, and 4.6, in the first 60 min after placental expulsion, were reported (52, 57).

Masuzawa et al. is the only study that investigated postpartum uterine contraction duration using TOCO (Table 2C) (56). Contraction duration was defined as the duration in seconds from the point where UA exceeded one-fifth of peak intensity above baseline, to the point where it dropped below this threshold. During the third stage of labor mean contraction duration was 54.4 ± 20.9 s. The duration of the contractions became longer over time with 83.3 ± 11.2 s in the first hour after placental expulsion and 102.7 ± 25.9 in the second hour after placental expulsion.

#### Uterine activity

The different studies measured UA across varying postpartum time frames using TOCO, IUPC or EHG. However, inconsistencies in definitions, ranges and measurement units, limited comparability.

Baseline UA was reported in six studies (Table 2D) (41, 44–46, 57, 62). Baseline UA measured with IUPC after childbirth was reported ≤ 15 mmHg (44–46). However, participants in the study of Marx et al. received analgesia and UA was measured with IUPC after placental expulsion. The authors reported higher baseline values with a range between 22.5 ± 2.8 and 26.7 ± 4.0 mmHg (62).

The intensity (i.e., maximum amplitude) of the UA was reported in eight studies (Table 2E) (37, 41, 44, 46, 56, 58, 59, 62). Various units for uterine intensity were reported, such as Torr (62), mmHg (44, 45, 56, 58, 59, 62), arbitrary units (a.u.) (41) and velocity squared (V^2^) (37). In three studies, the mean uterine intensity during the third stage of labor was measured using IUPC or TOCO. Comparable values of 58.0 ± 30.7, 51.7 ± 26.2 and 56.1 ± 13.3 mmHg were described (56, 58, 59). After placental expulsion, mean values between 69 and 110 mmHg were reported, but various cut-off points for maximum amplitudes were described (100–350 mmHg) (44, 45, 62). Furthermore, Diaz-Martinez et al. are the only researchers that compared postpartum uterine electrical activity between VD and CD. They found more frequent and intense UA after VD compared to CD (38).

Cumulative UA was reported in eight studies (Table 2F) (41, 44–46, 49, 53, 54, 60) and defined as total UA or Montevideo Units (MVU). First, total UA (i.e., total area under the curve (AUC), capturing intensity, frequency and duration of contractions) was presented in mW*s, kPas*s or in a.u. Different stimuli led to an increase in cumulative UA. Four studies showed an increased cumulative UA after the administration of uterotonics or after breastfeeding (44). First, Forman et al. showed an increased UA of 395 ± 38.2 to 648 ± 62.2 MVU following oxytocin IV administration of 20 mIU/min (45). Second, in women who received PGF_2a_ IV (35 μg/min), mean UA increased from 336 ± 41.5 MVU and reached their maximum 70 min after the start of infusion of 676 ± 79.0 MVU (44). Both studies monitored UA directly after placental expulsion. However, the sample size of the oxytocin and PGF_2a_ groups were very small (n = 5). Third, a small study of Granström et al. included five participants with PPH after standard therapy with oxytocin and additional uterotonics. UA was measured with IUPC after placental expulsion, 10 min before the start of 15-methyl prostaglandin F_2a_ infusion until 60 min after infusion. The administration of 15-methyl prostaglandin F_2a_ resulted in an increase in UA (in both UCF and amplitude, values not exactly mentioned) and cessation of blood loss within (mean) 12.5 min (64). Fourth, Chua et al. showed increased postpartum UA after breastfeeding and nipple stimulation in the period after placental expulsion (49). A reduction of cumulative UA was seen over time in all participants (44). In addition, Mas-Cabo et al. conducted a retrospective analysis of postpartum UA during the first 3 h after spontaneous VD in eight term patients. The authors reported a shift from high-intensity, coordinated UA to lower-intensity, more complex and irregular UA postpartum (36).

### What is known about the pathophysiology of postpartum UA?

Little is known about the relation of postpartum UA and TBL postpartum. Of all included studies, only eight described TBL postpartum (40, 41, 50, 56, 57, 59, 64, 65). The majority of these studies either excluded participants with HPP risk factors or focused on the effects of interventions or medication on TBL without describing the UA. TBL was measured in three different ways: visual inspection (65), colorimetric measurement of hemoglobin (50), or blood loss weighing (41, 56, 57, 59). In two studies, TBL measurement was not documented (40, 64).

The studies of Thijssen et al. and Frenken et al. were the only two studies which compared postpartum UA in women with and without PPH (40, 41). They both used EHG as monitoring method. No distinctions were made in the statistical analysis between the different PPH etiologies due to the limited PPH sample sizes in the two studies. Thijssen et al. conducted a prospective multi-center study and included 91 term women with a previous CD and a BMI ≥ 30 kg/m^2^. The study described a lower median UCF/10 min after placental expulsion and > 1 h after childbirth in women who developed severe PPH (i.e., TBL > 1,000 mL) (n = 9, 1.8 for both time periods) compared to women without PPH (n = 82, 2.7 after placental expulsion and 2.2 > 1 h postpartum). However, differences were not statistically significant. Frenken et al. conducted a prospective explorative monocenter study with 25 participants and routine use of oxytocin postpartum. The results showed a moderate positive correlation between postpartum TBL and total AUC (r = 0.44), AUC from baseline (r = 0.43) and baseline tone (r = 0.43). These described parameters were also significantly higher in participants with PPH (n = 3) compared to participants without PPH (n = 22), which is in contrast with previous results (40, 50, 56, 65).

Granström et al. is the only study that exclusively enrolled participants with PPH. However, the authors investigated the effect of 15-methyl-prostaglandin F_2a_ on postpartum UA and did not focus on UA morphology (64). Values to quantify UA were not mentioned. Furthermore, they excluded participants with retained placental tissue.

### Postpartum monitoring methods

#### Intrauterine pressure catheter

In total, 16 studies used IUPC for postpartum monitoring (44–46, 49, 50, 52–55, 57–59, 62–65). Only the two studies of Schorn et al. monitored UA during the third stage of labor whereas all the other studies monitored UA after placental expulsion. The duration of postpartum monitoring after placental expulsion varied between 20 and 360 min (Figure 2). Ingemarsson et al. monitored 30–45 min after childbirth for a total duration of 150 min. Based on the timeframe, it could be assumed that monitoring was started after placental expulsion, although this was not specified (57).

#### Feasibility

Eight studies described the feasibility of postpartum monitoring with IUPC (44, 45, 50, 52, 57–59, 65). The insertion process and use of the IUPC catheter after placental expulsion is described as easy (50, 57, 65) and with minimal or no discomfort for participants (44, 57, 65). However, insertion had to be conducted by a trained investigator (44) and Egarter et al. described one unintended expulsion of the catheter during movement of the participant (46). The insertion procedure directly after childbirth could be more challenging because the placenta fills a relative large portion of the uterine cavity as the uterus reduces in size (59). No complications related to postpartum IUPC monitoring were reported (44, 50, 52, 65).

Three studies described signal quality of postpartum UA, obtained by IUPC. Choo et al. described a reliable and good-quality signal (65). In contrast, Schorn et al. reported tracings of good quality in 36.5% of participants in one study and 60.0% in another study (58, 59). In the latter study, 36.0% of the tracings showed no obvious contractions. Whether uterine contractions were absent during the recorded period or whether the location or position of the IUPC made it difficult to detect contractions, is uncertain.

##### Electrohysterography

**Figure 4:**
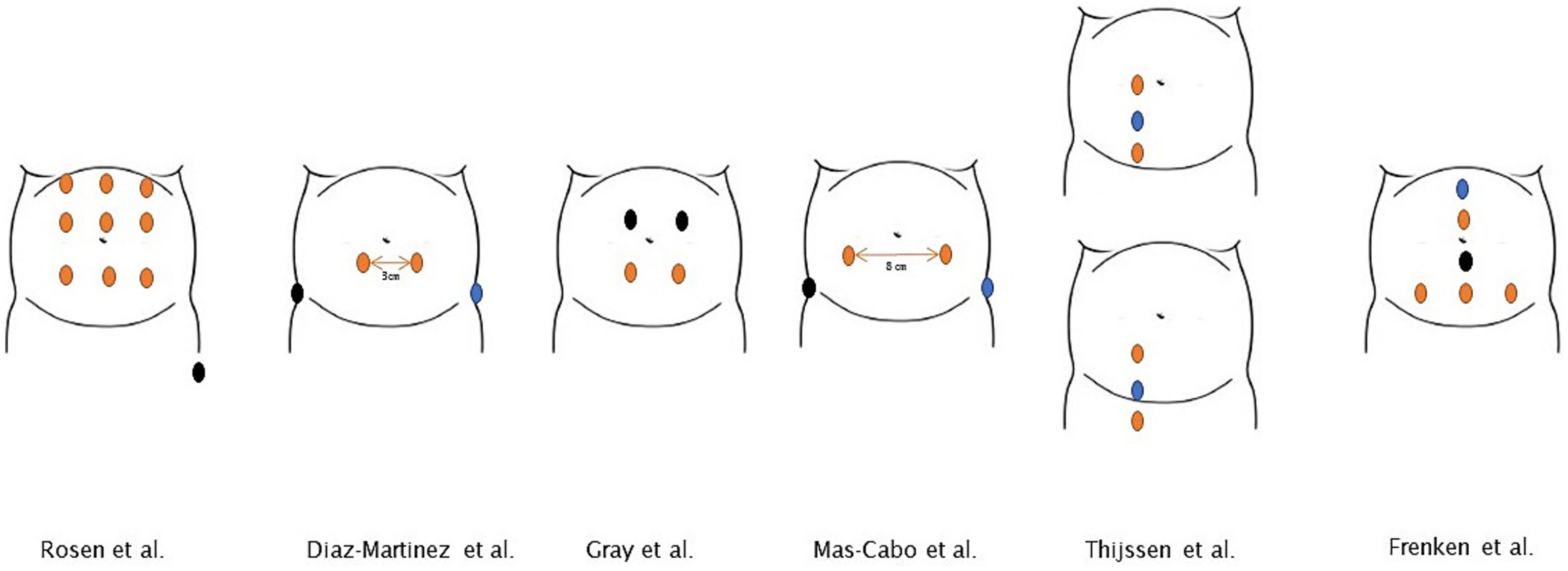
Various electrode positions for monitoring postpartum uterine activity. Abdominal electrode configurations across studies included in this review. Blue dot: bias/ground electrode (position not described in Gray et al.). Black dot: reference electrode. Orange dot: normal electrode.

In total, six studies used EHG for postpartum monitoring (36–38, 40, 60, 67). Over the past 5 years, this method has been the sole reported technique employed for postpartum monitoring. In contrast to IUPC monitoring, four out of six studies initiated EHG monitoring during the intrapartum period which was then continued postpartum (37, 40, 41, 60). The duration of EHG monitoring varied between 30 and ≥ 60 min. Comparison of the results is challenging due to variations in number of electrodes, interelectrode distance, position of the electrodes and UA post-processing methods. The different positions of the electrodes in the studies are presented in Figure 4 (36–38, 40, 41, 60).

#### Feasibility

Before attachment of the electrodes, abdominal skin preparation is required (38, 40, 41). This includes washing of the skin with water and soap, followed by gentle abrasion to optimize skin impedance. Thijssen et al. and Frenken et al. aimed for skin impedance values < 5 kΩ, whereas other studies did not mention a target value (40, 41). No studies reported complications while using EHG. Furthermore, 72.9% of the participants experienced no discomfort while using EHG monitoring during the third stage postpartum and after placental expulsion (40). In total, 95.5% of the participants recommend EHG to other patients. EHG was not recommended by the other 4.5% due to skin irritation or more accurate registration using external TOCO before patch placement (40).

Thijssen et al. concluded that readability of EHG tracings (visually assessed) reduced after childbirth. The readability of tracings was higher in women where the patch was positioned lower on the abdomen compared to participants with a higher patch position (40). Frenken et al. excluded 3 out of 28 participants from data analysis because of unavailable EHG tracings, defined as ≥ 6 min consecutive signal loss in the first 30 min postpartum (41).

#### Uterine activity analysis

All the studies used software to annotate UA. Thijssen et al. used visual annotation of contractions, whereas most of the studies used automatic whole-window analysis. In addition, motion artifacts were visually removed by experts (36, 38, 41). Thijssen et al. reported that most artifacts per hour occur during childbirth until placental expulsion (14.8 artifacts per hour). The least artifacts (0 per hour) were > 1 h postpartum. The number of artifacts in Thijssen et al. did not significantly differ across the two postpartum patch positions (27).

##### External tocodynamometry

Only Masuzawa et al. used TOCO as postpartum monitoring method (56). UA was monitored for a period of 120 min directly after placental expulsion. While the application of external TOCO was outlined, no information was provided regarding its practical implementation of potential challenges and complications. Additionally, none of the women expressed any discomfort regarding UA monitoring. Data of 17 women with a mean BMI of 19.1 kg/m^2^ were available for analysis. The number of participants eliminated from analysis due to failed registrations remains unclear. Moreover, the authors indicated that postpartum UA may be unidentifiable at times. However, it remained unclear if women had no contractions or if TOCO was unable to measure contractions adequately.

### Effect of medication on postpartum uterine activity

#### Uterotonic medication

Chong et al. measured UA with IUPC after placental expulsion and showed the most rapid and highest uterotonic effect of oral solution of misoprostol 400 μg compared to vaginal or rectal routes (54). Another study of Chong et al. showed a comparable uterotonic effect, measured with IUPC after placental expulsion, between misoprostol 400 μg and syntometrine (oxytocin/ergometrine) 1 mL IM, monitored during 30 min after administration (53). However, 200, 400 and 600 μg sublingual misoprostol resulted in significantly lower (*p* = 0.008) intrauterine pressures compared to 10 IU oxytocine IM in the first 10 min after placental expulsion. However, the opposite effect is visible after a longer period of time: the intrauterine pressures after all these misoprostol dosages were significantly higher 50–120 min after placental expulsion when compared to oxytocin administration (p = 0.008).

Amsalem et al. compared the effect of a high-dose oxytocin 10 IU IM with low-dose carbetocin 30 μg on postpartum UA after placental expulsion, measured for 240–360 min with IUPC. The study showed that low-dose carbetocin led to higher UA, with higher contraction frequency, duration and peak amplitude, compared to high-dose IV oxytocin (52).

Both studies of Forman et al. described the effect of 30 mg oral nifedipine on (1) spontaneous UA, (2) methylergometrine-induced UA, (3) oxytocin-induced UA and (4) PGF_2a_-induced UA, during the postpartum period (44, 45). UA was measured with IUPC. They concluded that nifedipine reduced UA in all four occasions. Furthermore, they provided a description of the effect of the uterotonics methylergometrine and prostaglandin F_2a_ on UA, before nifedipine was administrated. Methylergometrine 0.15 mg IV increased mean UA from 420 MVU to approximately 1,050 MVU. In addition, UCF increased and irregular contractions developed into regular contractions, and sometimes baseline tone elevated.

Besides the effect of various uterotonics on postpartum UA, a few studies investigated the effect of other medication on postpartum UA. Both studies of Marx et al. investigated the effect of different anesthetics on postpartum UA after placental expulsion by participants under analgesia (62, 63). Low concentrations of ketamine (25 mg), halothane (0.5 vol%) and enflurance (1.0 vol%) had no effect on postpartum UA. In contrast, higher concentrations of ketamine (50, 75 and 100 mg) increased postpartum UCF whereas higher concentrations of halothane (0.75 vol%) and enflurance (1.5 vol%) decreased UCF and UCI. Furthermore, the study of Egarter et al. showed a reduction in UA (baseline tone, frequency and intensity) after the administration of Pindolol (0.8 mg IV and 1.6 mg IV respectively) (46). In addition, the study of Ingemarsson et al. reported a reduction in UA after administration of 0.5 mg and 1 mg isradipine, with a greater reduction in UA with a higher dose of isradipine (57).

## Discussion

### Main findings

This is the first scoping review that outlines the relevant literature on postpartum UA and its continuous monitoring methods. It aimed to address four key questions regarding postpartum monitoring. The findings are summarized below.

1 What is known about postpartum UA?

Limited knowledge exists regarding postpartum UA. According to our assessment, comparability of findings is complicated by various monitoring methods, periods, durations, cut-off points of maximal amplitude and values to measure UA. An important observation was that mean UCF postpartum ranged between 2.4–2.8 contractions/10 min without uterotonics and between 3.7–4.6/10 min with uterotonics. Furthermore, notable reductions in UCF and UA intensity and an increase in UCI were observed over time during the postpartum period. Additionally, UA can be increased by other factors such as uterotonics and breastfeeding. Nonetheless, little is known about postpartum contraction duration over time.

2 What is known about the pathophysiology of postpartum UA?

Only eight studies have investigated the pathophysiology of postpartum UA, of which most focused primarily on the relationship between UA and TBL (40, 41, 50, 56, 57, 59, 64, 65). The majority excluded participants with HPP risk factors. Studies conducted prior to 2020, measured UA with IUPC or TOCO, report no significant correlation between UA and TBL. However, a small study using EHG conducted in 2024, cautiously suggest a positive relationship (41). These findings should be interpreted as hypothesis-generating. Improved uterine analysis techniques were applied due to the low PPH rates in the study groups, a knowledge gap exist in the description of UA in participants with (different causes of) PPH.

3 Which methods can be used to monitor UA postpartum?

Three methods were used to monitor postpartum UA. Based on the findings, external TOCO appears less suitable for postpartum monitoring, as only one out of 29 studies reported its use (56). Furthermore, it remains unclear whether identifiable contractions were actually recorded. Instead, monitoring UA with IUPC seems particularly suitable after placental expulsion because of the risk of IUPC expulsing during childbirth or placental expulsion. Given that the most recent article with IUPC for postpartum UA monitoring method was published in 2012, its relevance to current clinical practice appears to be limited (58). In recent years, EHG has emerged as promising non-invasive method for postpartum UA monitoring. Intrapartum EHG monitoring can easily be continued for postpartum monitoring (38, 40, 41, 60). This non-invasive technique eliminates issues of repositioning or expulsion. However, the absence of standardized methods, generalized, large data bases and the interpretation of postpartum EHG currently limits the adoption in clinical care.

1. 4 What is the effect of postpartum uterotonic medication on UA?

Only studies utilizing IUPC monitoring have examined the effect of various uterotonic medications on postpartum UA parameters (44–46, 52–54, 57, 62, 63). Changes in these parameters reveal the impact of uterotonics. It emphasizes the need for further research on the impact of various uterotonics on UA parameters.

### Strengths and limitations

This scoping review has several strengths. First, it is the first study to outline all available literature on postpartum UA, providing insight into the key terms and knowledge gaps in this field. Second, a transparent and secured methodology for scoping review was employed. Third, a complete and unbiased literature selection was conducted, as no filter restrictions were applied. Fourth, patients served as their own controls in most of the studies, which minimized the impact of biological and individual variations. Finally, although a risk of bias assessment is not required for scoping reviews, the studies were nonetheless evaluated for quality. Therefore, only results with a low or moderate risk of bias were reported. However, certain limitations should be acknowledged. First, the studies are difficult to compare due to differences in, e.g., population, monitoring methods, timing and duration of monitoring, study design, UA parameters, UA analysis techniques as well as types and dosages of medication used postpartum. In addition, also comparison between studies utilizing EHG was complicated because of variation in electrode size, electrode positions and inter-electrode distance. Second, the visual interpretation and visual cutoff values of UA may be subjective ad potentially affect the comparability of data. Third, the sample sizes of the included studies were small, especially for studies with PPH inclusions. Besides, studies often excluded participants with PPH risk factors, reducing its applicability to the general population. Only six studies monitored UA directly after childbirth, whereas in this period complications such as excessive blood loss frequently occur (37, 38, 40, 41, 58–60). As a consequence, caregivers often prescribe preventive medication in order to reduce the incidence of PPH due to uterine atony or retained placental tissue. Furthermore, the majority of studies focused on the effect of postpartum medication on UA. In these studies, medication was often administered after placental expulsion (and start of study recordings), which generally does not reflect clinical practice where medication is often administered before placental expulsion. Another limitation is that we did not include studies utilizing other monitoring methods, such as ultrasound monitoring postpartum. However, we chose not to include these studies as we were interested in continuous monitoring methods postpartum and ultrasound seemed less suitable for this goal (68).

### Comparison with existing literature

Masuzawa et al. was the only study that measured postpartum UA with TOCO. The authors reported UCI, UA intensity and duration of UC. However, with the current state of knowledge, TOCO lacks the ability of adequate monitoring of UA intensity and duration of contractions (69).

This scoping review presents an overview of the current state of postpartum monitoring, contributing to addressing the knowledge gaps and highlighting topics that require further investigation. Moreover, up to our knowledge, this is the first systematic scoping review regard to uterine activity monitoring during the postpartum period.

The identified knowledge gaps include the need for: (1) more detailed information regarding UA directly after childbirth and around placental expulsion, (2) description of UA in women with PPH, and (3) comparison of UA in women with PPH originating from different causes such as uterine atony and retained placental tissue. By understanding the physiology of postpartum UA, the pathophysiological UA can be recognized earlier. This may contribute to earlier prediction of PPH and improve its preventions and successful management.

Our results indicate that both IUPC and EHG are accurate for postpartum monitoring. However, the insertion procedure of IUPC after childbirth is complicated and IUPC can be expelled during childbirth or placental expulsion (58, 59). Although EHG enables continuous monitoring immediately postpartum, little is known about the optimal patch position for postpartum monitoring. In addition to the UA parameters measured with IUPC (e.g., frequency and intensity of contractions), our result indicate that specific EHG parameters, including spectral, temporal and non-linear features, can also be assessed for understanding the postpartum UA. Furthermore, there is limited data on the experiences of patients and healthcare providers regarding the value of postpartum monitoring, and relatively little information about the signal quality postpartum.

Future research should aim to improve our understanding of the physiology of UA patterns and pathophysiological deviations on a large scale. Study populations should reflect the general obstetric population, without excluding individuals at risk for PPH. To enable large-scale clinical implementation, the development of computer-based UA algorithms and automated artifact detection systems is essential. There is need for a uniform and objective method to monitor postpartum UA. In addition, standardized definitions and units should be incorporated to quantify postpartum UA consistently.

As a concrete next step, we propose a minimal reporting framework for postpartum EHG studies. To improve comparability across the studies, future research should report: (1) electrode configuration including number of electrodes, inter-electrode distance and anatomical landmarks (2) skin preparation protocol and achieved impedance values; (3) signal acquisition parameters (sampling frequency, filtering bandwidth); (4) contraction annotation method (visual vs. automated, window length, artifact rejection criteria); (5) definition of baseline UA, for example defined as the 10th percentile of EHG signal within a fixed time window; and (6) definition of cumulative UA (e.g., AUC corrected for baseline). Monitoring UA during at least the first 60 min postpartum, including the third stage of labor and the period after placental expulsion, is recommended. Objective quantification of vaginal blood loss should be incorporated to allow meaningful correlation with UA parameters.

## Conclusion and implications

Current evidence on objective monitoring of postpartum UA reveals a lack of established reference values for the postpartum physiology, as well as insufficient data regarding UA characteristics in patients with (various etiologies of) PPH. However, a reduction in UCF (values of UCF per 10 min between 2.4 and 2.8 without uterotonics and between 3.7 and 4.6 with oxytocin) and UA intensity is observed over time during the postpartum stage. EHG is a non-invasive technique that holds significant potential to enhance our understanding of UA (patho)physiology.

## Data Availability

The original contributions presented in the study are included in the article/Supplementary material, further inquiries can be directed to the corresponding author.
