# Supplementary material for: Objective monitoring of postpartum uterine activity: a systematic scoping review
Source: Front Med (Lausanne). 2026 Mar 5;13:1703494. doi: 10.3389/fmed.2026.1703494 (PMC12999783; doi:10.3389/fmed.2026.1703494)
Supplement: Supplementary file 1 [file Data_Sheet_1.docx]

|  | | | | **Uterine contraction interval** | | | |
| --- | --- | --- | --- | --- | --- | --- | --- |
| **First Author,  Year** | **N** | **Monitoring method** | **Profylactic oxytocin postpartum** | | **Timing** | **Definition & unit** | **Result: mean ± SD (range lowest- highest value)** |
| Schorn, 2009 | 41 | IUPC | Conform local protocol | | Third stage | Onset-to-onset time between contractions in minutes | 2.75±0.29 (1-5) |
| Schorn, 2012 | 36 | IUPC | 18/36 participants received oxytocin conform local protocol | | Third stage | Onset-to-onset time between contractions in minutes | 2.6±0.9 (1-5) |
| Masuzawa, 2017 | 17 | TOCO | 2/17 participants received 5 IU oxytocin IV | | Third stage | Minutes between the first contraction peak to the second contraction peak | 2.4 ± 0.9 |
|  |  |  |  |  | First 60 min after placental expulsion |  | 4.2 ± 0.7 |
|  |  |  |  |  | 60-120 min after placental expulsion |  | 7.9 ± 2.1 |

**A.**

Abbreviations: IU: international units, IUPC: intrauterine pressure catheter, IV: intravenous, Min: minutes, SD: standard deviation, TOCO: external tocodynamometry

**B.**

| **Uterine contraction frequency per ten minutes** | | | | | |
| --- | --- | --- | --- | --- | --- |
| **First Author,  Year** | **N** | **Monitoring method** | **Profylactic oxytocin postpartum** | **Timing** | **Result: mean ± SD (range lowest- highest value)** |
| Marx, 1979^#^ | 22 | IUPC | None | First 10 min after placental expulsion | (2.4±0.2 - 2.8±0.5) |
| Forman, 1982 | 17 | IUPC | None | First 10 min after placental expulsion | 2.6 |
| Egarter, 1988 | 16 | IUPC | None | First 10 min after placental expulsion | 2.6±0.6 |
| Ingemarsson, 1989* | 34 | IUPC | None | Duration of 30 min, 30-45 after childbirth | 2.7 |
|  |  |  |  | Duration of 15 min, 45-60 min after childbirth | 2.4 |
|  |  |  | 12.5 mU/min IV | Duration of 30 min, 30-45 after childbirth | 3.7 |
|  |  |  |  | Duration of 120 min, 60-75 min after childbirth | 2.1 |
| Amsalem, 2014 | 12 | IUPC | 10 IU oxytocin IM | First 60 min after placental expulsion | 4.6 |
|  |  |  |  | 60-240 min after placental expulsion | 2.1 |
| Thijssen, 2023** | 91 | EHG | 90% received oxytocin conform local protocol | Third stage | 3.7 |
|  |  |  |  | 60 min postpartum | 2.7 |
|  |  |  |  | 120 min postpartum | 2.2 |

Abbreviations: EHG: electrohysterography, IM: intramuscular IU: international units, IUPC: intrauterine pressure catheter, IV: intravenous, Min: minutes, mU: milli-Units, SD: standard deviation
^#^ All participants have an pundendal block with or without nitrous oxide-oxygen analgesia or with nitrous oxide-oxygen analgesia alone
^*^ Uterine contraction frequency is calculated from 15 to 10 minutes
** Uterine contraction frequency is calculated from 60 to 10 minutes
 **C.**

|  | **Uterine contraction duration in seconds** | | | | | |
| --- | --- | --- | --- | --- | --- | --- |
| **First Author,  Year** | **N** | **Monitoring method** | **Profylactic oxytocin postpartum** | **Definition & unit** | **Timing** | **Result: mean ± SD** |
| Masuzawa, 2017 | 17 | TOCO | 2/17 participants received 5 IU oxytocin IV | Duration in seconds at the point of one-fifth intensity from baseline | Third stage | 54.4 ± 20.9 |
|  |  |  |  |  | First 60 min after placental expulsion | 83.3 ± 11.2 |
|  |  |  |  |  | 60-120 min after placental expulsion | 102.7 ± 25.9 |

Abbreviations: IU: international units, IV: intravenous, Min: minutes, SD: standard deviation, TOCO: external tocodynamometry **D.**

| **Baseline uterine activity^***^** | | | | | | | |
| --- | --- | --- | --- | --- | --- | --- | --- |
|  |  |  |  |  | **Baseline UA^***^** | | |
| **First Author,  Year** | **N** | **Monitoring method** | **Profylactic oxytocin postpartum** | **Timing** | **Definition** | **Unit** | **Result: mean ± SD or median with [interquartile range]**  **(range lowest- highest value)** |
| Marx, 1979 ^#, **^ | 22 | IUPC | None | Unknown time period after placental expulsion, duration 10 min | No definition for resting pressure | mmHg | (22.5±2.8 - 26.7±4.0) |
| Forman, 1982 | 17 | IUPC | None | First 10 min after childbirth | No definition for basic tone | mmHg | <10 |
| Forman, 1982 | 19 | IUPC | None | First 20-30 min after childbirth | No definition for basic tone | mmHg | 10 |
| Egarter, 1988 | 16 | IUPC | None | First 10 min after childbirth | No definition for basal tone | mmHg | 7.9±3.4 |
| Ingemarsson, 1989 | 34 | IUPC | None | Duration of 30 min, 30-45 min after childbirth | No definition for basal tone | mmHg | 10-15 |
| Frenken, 2025 | 23 | EHG | 5IU oxytocin IV | First 30 min after childbirth | Baseline: 10^th^ percentile of all UA in 30 min | Arbitrary units | 12.1 [8.1 – 23.1] |

Abbreviations: EHG: electrohysterography, IU: international units, IUPC: intrauterine pressure catheter, IV: intravenous, Min: minutes, mmHg: millimetres of mercury, SD: standard deviation, UA: uterine activity
^#^ All participants have an pundendal block with or without nitrous oxide-oxygen analgesia or with nitrous oxide-oxygen analgesia alone
^**^ Resting pressure is calculated from Torr to mmHg
^***^ Maximum intensity of amplitude is not described, only 350mmhg in both studies of Forman et al.

**E.**

| **Uterine intensity (i.e. maximum amplitude)** | | | | | | | |
| --- | --- | --- | --- | --- | --- | --- | --- |
| **First Author,  Year** | **N** | **Monitoring method** | **Profylactic oxytocin postpartum** | **Timing** | **Range** | **Unit** | **Result: mean ± SD or median with [interquartile range]**  **(range lowest- highest value)** |
| Marx, 1979^#^ | 22 | IUPC | None | First 10 min after placental expulsion | 0-260 | mmHg | (93.3±15.4 - 110.0±10.7) |
| Forman, 1982 | 17 | IUPC | None | First 10 min after childbirth | 110-350 | mmHg | 160 |
| Egarter, 1988 | 16 | IUPC | None | First 10 min after childbirth | Not described | mmHg | 69.1±26.7 |
| Schorn, 2009 | 41 | IUPC | Conform local protocol | Third stage | 20-100 | mmHg | 51.7±26.2 |
| Schorn, 2012 | 36 | IUPC | 18/36 participants received oxytocin conform local protocol | Third stage | 20-100 | mmHg | 58.0±30.7 |
| Masuzawa, 2017 | 17 | TOCO | 2/17 participants received 5 IU oxytocin IV | Third stage | Not described | mmHg | 56.1±13.3 |
|  |  |  |  | First 60 min after placental expulsion | Not described | mmHg | 50.8±18.1 |
|  |  |  |  | 60-120 min after placental expulsion | Not described | mmHg | 50.8±18.1 |
| Gray, 2020 | 28 | EHG | Not described | Third stage | Not described | V^2^E^-8^ | 7 |
| Frenken, 2025 | 23 | EHG | 5IU oxytocin IV | First 30 min after childbirth | Not described | Arbitrary units | 303.0 [176.4 – 761.1] |

Abbreviations: EHG: electrohysterography, IU: international units, IUPC: intrauterine pressure catheter, IV: intravenous, Min: minutes, mmHg: millimetres of mercury, SD: standard deviation, TOCO: external tocodynamometry, V: velocity
^#^ All participants have an pundendal block with or without nitrous oxide-oxygen analgesia or with nitrous oxide-oxygen analgesia alone

**F.**

| **Cumulative uterine activity** | | | | | | | |
| --- | --- | --- | --- | --- | --- | --- | --- |
| **First Author,  Year** | **N** | **Monitoring method** | **Profylactic oxytocin postpartum** | **Timing** | **Definition** | **Unit** | **Result: mean ± SD or median with [interquartile range]**  **(range lowest- highest value)** |
| Forman, 1982*** | 17 | IUPC | None | First 10 min after childbirth | Not described | MVU | 420 |
| Forman, 1982*** | 19 | IUPC | None | First 45-70 min after placental expulsion | Not described | MVU | 395±38.2 |
| Egarter, 1988 | 16 | IUPC | None | First 10 min after childbirth | Not described | MVU | 176.6 ± 76.5 |
| Chua, 1994 | 11 | IUPC | None | 30-60 min after placental expulsion | Average UA of two 15-minutes values. Active pressure per contraction: manual recording of the peak pressure minus the average basal tone in a 15 minute window. | MVU | (5-1313), mean value 612 MVU per 15 min, i.e. 408 per 10 min |
| Chong, 2001 | 57 | IUPC | Conform local protocol | First 30 min after placental expulsion | \| Baseline cumulative uterine activity over 30 min \| \| --- \| | kPA*s | (5358±1877 - 7216±2776) |
| Chong, 2004 | 50 | IUPC | Conform local protocol | First 30 min after placental expulsion | \| Baseline cumulative uterine activity over 30 min \| \| --- \| | kPA*s | 3772.5±3320.7 - 5806.0 ± 3610.5) |
| Rosen, 2014 | 44 | EHG | None | Third stage | Not described | mW*s | 3.4 ± 0.6 |
| Frenken, 2025 | 23 | EHG | 5IU oxytocin IV | First 30 min after childbirth | Total area under the contraction curve | Arbitrary units | 96486.1 [62394.5 - 139526.6] |

Abbreviations: EHG: electrohysterography, IU: international units, IUPC: intrauterine pressure catheter, IV: intravenous, KPA*s: kilopascal-seconds, Min: minutes, MVU: Montevideo Units, mW*S: milliwatt-seconds
*** Maximum intensity of amplitude is not described, only 350mmhg in both studies of Forman et al.
